# Comparing Traditional and in Motion Nd:YAG Laser in Hair Removal: A Prospective Study

**DOI:** 10.3390/medicina58091205

**Published:** 2022-09-02

**Authors:** Steven Paul Nistico, Luigi Bennardo, Stefano Bennardo, Miriam Marigliano, Elena Zappia, Martina Silvestri, Giovanni Cannarozzo

**Affiliations:** 1 Department of Health Sciences, Magna Graecia University, 88100 Catanzaro, Italy; 2 Department of Dermatology, Sapienza University, 00185 Rome, Italy

**Keywords:** hair removal, Nd:YAG laser, in motion technology

## Abstract

*Background and Objectives*: Hair removal is a common cosmetic problem interesting more and more patients nowadays. Various laser treatments are currently available. Alexandrite and Nd:YAG laser are the most effective procedures in lighter and darker skin phototypes, respectively. *Materials and Methods*: A total of 40 patients seeking hair removal in one or more body areas with skin phototypes 2–6 was recruited to perform this study. Patients were divided into two groups. One group was treated with the standard Nd:YAG hair removal procedure, while the other group was treated with a new “in motion” Nd:YAG technology. Results and hair removal rates were evaluated six months after the last treatment. *Results*: Out of 40 patients treated, all patients experienced hair reduction. No statistically significant difference in hair removal was noted between the two groups; however, a statistically significant reduction in pain during the procedure was observed in the group treated with the “in motion” technique. Conclusions: While traditional and “in motion” Nd:YAG techniques have similar result outcomes in hair removal, the “in motion” technology seems to guarantee a better safety profile compared with the traditional technique. A more extensive clinical study will be necessary to confirm our study’s results.

## 1. Introduction

The abundance of hair is a common cosmetic problem, and patients seeking hair removal are becoming more frequent in medical practice [1,2]. Laser and light sources represent the mainstay in managing this condition [3]. Among laser systems currently used in hair removal management, 755 nm alexandrite lasers are usually preferred for lighter phototypes, while Nd:YAG lasers are used for darker phototypes [4,5]. Nd:YAG laser emits in the infrared spectrum at 1064 nm. According to its emission modality, this device can be used to manage different dermatological conditions. When emitting in pulsed mode, these types of lasers are used in the removal of hyperpigmentations and tattoos [6,7,8,9]. In continuous mode, this laser is instead used to manage vascular lesions and hair removal [10,11]. The longer wavelength of the Nd:YAG allows for less epidermal melanin absorption. Patients can tolerate higher fluences with minimal adverse events such as epidermal burn or dyspigmentation. The long-pulse Nd:YAG laser did not demonstrate significant long-term adverse events at high fluences (50, 80, and 100 J/cm^2^) when treating skin phototypes up to four, with only a tiny percentage of patients treated at 100 J/cm^2^ developing nonscarring blisters. Greater fluence did not result in more significant hair reduction, with similar efficacy in hair reduction in the two treatment groups (27–29%) at a 3-month follow-up [12]. In this work, we compare a new “in motion” technology with the traditional Nd:YAG laser in managing hair removal.

## 2. Materials and Methods

Forty patients with phototypes 2–6 presenting for hair removal were consecutively enrolled at the Unit of Dermatology of Magna Graecia University (Catanzaro, Italy) and at private practice (Florence, Italy). Patients reporting hypersensitivity to light or reporting the use of sulfonamides, phenothiazines, and contraceptives, being pregnant, breastfeeding, or with malignant tumors were excluded from the study. All patients signed informed consent on the risk of the procedure. Patients were divided into two groups. The first group underwent traditional therapy for hair removal with Nd:YAG laser (MotusAY, DEKA M.E.L.A., Calenzano, Italy) (Fluence: 30–40 J/cm^2^ Pulse duration: 10–20 ms Spot: 10–15 mm). Six treatments were performed with an interval between sessions of 4–8 weeks according to body area treated (face, groin, legs, or trunk).

**Figure 1:**
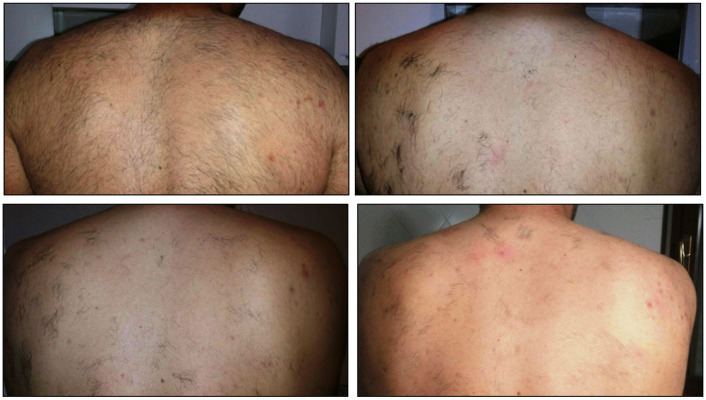
Patient 27 treated with the “in motion” technique before treatment (upper left picture) after two sessions (upper right picture), four sessions (lower left picture), and six sessions (lower right picture).

The second group was treated with the “in motion” Nd:YAG handpiece of the same system (MoveoHR, MotusAY, DEKA M.E.L.A., Calenzano, Italy) with the following parameters: fluences ranging from 3 to 8 J/cm^2^, dose ranging from 1.5 to 3.5 KJ, and frequency ranging from 3 to 5 Hz, according to the endpoint (the development of perifollicular erythema). For the “in motion” technique, the user continuously moved the handpiece in a slow linear/circular motion, creating an area of 10 × 10 cm and executing multiple back-and-forth passes. The interested areas were shaved 48 h before each treatment. Before every treatment session, the same physician (S.N.) evaluated all patients. Hair reduction rate (R%) was quantified utilizing the following formula: ((the hair quantity before the first laser treatment–the hair quantity after the current laser treatment)/the hair quantity before the first laser treatment) × 100. Response to both treatments was assessed six months after the last treatment session. Immediately after each laser session, the same physician evaluated side effects, using a five-point scale to evaluate erythema and the presence of first-, second-, or third-degree burns. A visual analogue scale from 0 to 10 at the end of each treatment was administered to the patients to evaluate pain. At the end of treatment, a visual analogue scale about treatment satisfaction (from 1 to 8) was administered to the subjects. Student’s t test for paired data was used to compare the results obtained between groups. Statistica14.0 (TIBCO Software, Palo Alto, CA, USA) software was used for data analysis (mean, standard deviations, and rate calculations) (Figure 1).

## 3. Results

The patients were divided into two groups, not differing in sex distribution, age, or phototype (*p* > 0.05). Both groups reported a hair reduction of almost 85% (82.2% ± 6.9% for group one and 79% ± 5.4 for group two), with no statistical difference (*p* = 0.16). Patients of both groups reported a high degree of satisfaction (5.95 ± 1.09 for group one and 6.15 ± 1 for group two) with no statistically significant difference (*p* > 0.05). Group one reported a higher level of erythema after treatment (2.15 ± 0.99 vs. 1.65 ± 0.67), but this difference was not statistically significant (*p* > 0.05). Group one reported a higher level of pain during the procedures (6.05 ± 1.39 vs. 3.55 ± 1.15). This result was statistically significant (*p* < 0.001). No severe side effects were reported; however, in the traditional Nd:YAG group; one patient experienced a first-degree burn. All patients’ characteristics are reported in Table 1.

## 4. Discussion

The effectiveness of hair removal with the long-pulsed 1064 Nd:YAG laser was described in the late 1990s when Nd:YAG was proposed, first in pulsed mode and then in continuous mode for hair removal [13,14,15]. Since then, many studies, including the current study, have confirmed this device’s efficacy and safety profile, reporting a mean hair reduction of up to 80%. Ismail reported that at the six-month follow-up after all the procedures, patients treated with long-pulsed Nd:YAG laser experienced a 79.4% decrease in hair count [16]. Other studies reported a decrease in hair count from 50 to 60%, but this variability may be associated with the researcher experience, as well as with a different treatment protocol [17,18]. Recently, short-pulsed Nd:YAG laser has been proposed for hair removal, with results that need further confirmation [19]. Our study confirmed good results in managing phototypes two and three, traditionally treated with other laser devices, showing overall good results. Pain was the patients’ most referred concern, but it was bearable during all the procedures when using the new “in motion” technology. Only one minor burn was reported, using the traditional technique, that spontaneously resolved without leaving any scar or dyspigmentation in a couple of weeks while applying an antibiotic cream. Containment of the side effects and the pain can be explained through the emission mode of the “in motion” technique. The technique, which uses a cooled handpiece, indeed, uses a minimal energy emission to reduce the pain sensation. The physician continuously moved the handpiece in a slow linear/circular motion inside an area of 100 square centimeters and executing multiple back-and-forth passes up to a defined follicular heat endpoint. By doing so, the areas are treated in a short length of time, the device is easier to use—as skin does not easily reach high temperatures associated with burns—and larger areas are treated more rapidly. This method allows a progressive increase of the target temperature, monitoring the cutaneous reactions, and being able to interrupt or modify the treatment at any time, thus minimizing the typical side effects of the traditional method, as already proposed for other typologies of lasers in hair removal [20].

## 5. Conclusions

Standard log pulsed Nd:YAG laser and Nd:YAG with Moveo emission have proven to be effective and safe technologies capable of achieving long-standing results in body hair removal in Fitzpatrick’s skin types 2–6. In our study, however, this new “in motion” technology, while being equally effective, has proven to be associated with less erythema, side effects, and only minor patient-perceived pain. Of course, further studies will be necessary to confirm our study results.

## Figures and Tables

**Table 1:** Patient characteristics.

Patient Number	Phototype	Sex	Body Location	Age	VAS Erythema	VAS Treatment Satisfaction	Hair Reduction Rate	Pain VAS (Mean)
Group 1 standard Nd:YAG								
1	3	F	Legs	21	3	6	86	6
2	5	M	Groins	32	1	7	87	7
3	2	F	Legs	26	3	5	80	7
4	5	F	Legs	41	4 (burn)	5	74	6
5	6	F	Face	29	2	5	96	8
6	5	M	Legs	19	2	6	85	6
7	5	F	Trunk	31	1	6	77	7
8	3	F	Groins	22	3	7	73	6
9	6	M	Legs	28	2	8	93	6
10	3	F	Face	48	4	4	79	8
11	5	F	Trunk	19	1	5	80	7
12	4	F	Legs	31	1	5	76	4
13	4	F	Legs	37	1	6	72	3
14	6	M	Face	34	2	5	90	5
15	4	F	Groins	47	3	6	87	6
16	6	F	Trunk	18	2	7	79	4
17	5	F	Legs	29	1	6	82	7
18	3	F	Groins	21	2	7	74	5
19	4	F	Legs	39	3	7	88	8
20	3	F	Trunk	45	2	6	86	5
Group 2 “in motion” Nd:YAG								
21	3	F	Legs	22	2	6	77	3
22	5	F	Groins	18	1	5	70	4
23	2	F	Face	45	1	6	82	3
24	5	F	Legs	48	1	6	83	2
25	6	M	Legs	26	2	6	78	6
26	5	F	Groins	39	2	7	86	3
27	5	M	Trunk	29	2	3	89	4
28	3	F	Legs	21	1	7	83	3
29	6	F	Face	26	3	7	74	2
30	3	F	Groins	48	1	6	71	6
31	5	F	Legs	39	2	7	77	4
32	4	M	Legs	36	2	6	70	5
33	4	F	Trunk	29	2	7	79	3
34	6	F	Face	22	3	7	74	4
35	4	F	Legs	19	2	4	86	4
36	6	F	Groins	23	1	7	77	2
37	5	F	Trunk	25	1	7	79	3
38	3	M	Groins	34	1	7	84	4
39	4	F	Legs	41	2	6	80	3
40	3	F	Legs	32	1	6	81	3

## Data Availability

Data are available from the corresponding author upon reasonable request.
